# *Terminalia chebula* Fructus Inhibits Migration and Proliferation of Vascular Smooth Muscle Cells and Production of Inflammatory Mediators in RAW 264.7

**DOI:** 10.1155/2015/502182

**Published:** 2015-02-16

**Authors:** Hyun-Ho Lee, Keshav Raj Paudel, Dong-Wook Kim

**Affiliations:** Department of Oriental Medicine Resources, Mokpo National University, Muan-gun, Jeonnam 534-729, Republic of Korea

## Abstract

Pathogenesis of atherosclerosis and neointima formation after angioplasty involves vascular smooth muscle cells (VSMCs) migration and proliferation followed by inflammatory responses mediated by recruited macrophages in the neointima. *Terminalia chebula* is widely used traditional medicine in Asia for its beneficial effects against cancer, diabetes, and bacterial infection. The study was designed to determine whether *Terminalia chebula* fructus water extract (TFW) suppresses VSMC migration and proliferation and inflammatory mediators production in macrophage (RAW 264.7). Our results showed that TFW possessed strong antioxidative effects in 1,1-diphenyl-2-picryl hydrazyl (DPPH) scavenging and lipid peroxidation assays. In addition, TFW reduced nitric oxide (NO) production, inducible nitric oxide synthase (iNOS), and cyclooxygenase-2 (COX-2) expression in RAW 264.7 cells. Also, TFW inhibited platelet-derived growth factor (PDGF-BB) induced VSMC migration as determined by wound healing and Boyden chamber assays. The antimigratory effect of TFW was due to its inhibitory effect on metalloproteinase-9 (MMP-9) expression, focal adhesion kinase (FAK) activation, and Rho-family of small GTPases (Cdc42 and RhoA) expression in VSMCs. Furthermore, TFW suppressed PDGF-BB induced VSMC proliferation by downregulation of mitogen activated protein kinases (MAPKs) signaling molecules. These results suggest that TFW could be a beneficial resource in the prevention of atherosclerosis.

## 1. Introduction

Pathogenesis of atherosclerosis and neointima formation after angioplasty can be described as the complex involvement of several growth factors including platelet-derived growth factor (PDGF-BB) and tumor necrosis factor alpha (TNF-*α*) that induce vascular smooth muscle cells (VSMCs) migration and proliferation [1, 2]. During the early stages of atherosclerosis or arterial wall injury, VSMCs may undergo transition from a contractile to a synthetic phenotype and begin proliferation in response to PDGF-BB, a potent growth factor and chemoattractant produced by vascular endothelial cells, platelets, VSMCs, and macrophages [3]. PDGF-BB and TNF-*α* have been reported to promote cell proliferation through mitogen activated protein kinases (MAPKs) and nuclear factor kappa B (NF-*κ*B) activation pathways [4]. The accumulation of VSMCs within the intima is the result of VSMC migration from media to intima in response to PDGF-BB [5]. VSMC migration from media to intima involves enzymatic action of matrix metalloproteinases (MMPs), specifically MMP-2 and MMP-9, that degrade extracellular matrix (ECM) thereby facilitating VSMC migration [6]. In addition, cytoskeleton remodeling, which is associated with focal adhesion kinase (FAK) phosphorylation and small GTPases activation, is required to generate the driving force for VSMC migration [7–9]. Therefore, inhibition of the molecular pathways responsible for VSMC proliferation and migration is a widely used strategy in the development of the drugs against atherosclerosis and restenosis.

Atherosclerotic progression follows inflammatory responses mediated by macrophages. The circulating monocytes are recruited and differentiated into macrophages in response to monocyte chemoattractant protein (MCP-1) [10]. Activation of these macrophages results in inducible nitric oxide synthase (iNOS) and cyclooxygenase (COX-2) synthesis [11]. Studies have shown proatherosclerotic role of iNOS that is caused by the massive production of nitric oxide (NO) and subsequent formation of peroxynitrite [12], a powerful oxidant, and can lead to oxidation of low density lipoproteins (LDL) [13]. Macrophages engulf oxidized LDL and turn into foam cells [14]. Prostaglandins produced by the action of COX-2 have been shown to be mitogenic, leading to cellular proliferation [15].

Most people in developing countries depend on complementary and alternative therapies, especially those derived from botanical origins. They use plants as a source of medicine for their primary health needs, because plants are considered less toxic, with fewer adverse effects than synthetic drugs [16]. Therefore, interest in natural remedies and therapeutic use of natural products for the prevention of atherosclerosis and associated inflammation has been growing.* Terminalia chebula*, which is naturally distributed to South East Asia, is one of the promising traditional medicines. Application of* T. chebula* has been widely accredited in Ayurveda [17]. Various forms of* T. chebula* extracts have been frequently applied as traditional medicines for their laxative, astringent, purgative, diuretic, antioxidant, antimicrobial, antidiabetic, and antimutagenic activity [18].* T. chebula* has been considered to promote health, immunity, and longevity [19]. However, to the best of our knowledge, no one has reported the beneficial effects of* T. chebula* in atherosclerosis and associated inflammation. Therefore, the aim of this study was to determine whether* T. chebula* possessed antiatherosclerotic action through modulating VSMC proliferation and migration and attenuates inflammatory responses mediated by RAW 264.7.

## 2. Materials and Methods

### 2.1. Reagents

MTT (3-[4,5-dimethylthiazol-2-yl]-2,5-diphenyl tetrazolium bromide), antibody to *β*-actin, and lipopolysaccharide (LPS) were purchased from Sigma (St Louis, MO, USA). The antibodies to phosphorylated ERK (pERK1/2), ERK1/2, pJNK, JNK, pFAK, and FAK were purchased from Cell Signaling Technology (Beverly, MA, USA), antibodies to RhoA, Cdc42, and GAPDH were purchased from Santa Cruz (Santa Cruz, CA, USA), and antibody to MMP-9 was obtained from Millipore (Billerica, MA, USA). Platelet-derived growth factor (PDGF-BB) and tumor necrosis factor alpha (TNF-*α*) were obtained from R & D systems (Minneapolis, USA). Nitrocellulose membrane and chemiluminescent for Western blotting were purchased from Bio-Rad (Richmond, CA, USA) and IMEGENEX (San Diego, CA, USA), respectively. All solvents, chemicals, and reagents were of analytical grade and purchased from Sigma-Aldrich unless otherwise specified.

### 2.2. Plant Material

Dried fruits from* T. chebula*, purchased from Muan province of Korea, were extracted with hot water for 3 times using soxhlet extractor. The extract was then filtered and evaporated under vacuum followed by lyophilization. The lyophilized powder (TFW) was resuspended in distilled water and filtered by a 0.22 *μ*m for use in cell culture. The yield of the extract was about 9% of the starting material. Total phenolic acid content in TFW was measured as described previously [20]. The polysaccharide and protein contents in TFW were assessed using the phenol-sulfuric acid method and Bio-Rad protein assay kit (Hercules, CA, USA), respectively. The tannin content was determined by Folin and Ciocalteu method and expressed as mg gallic acid equivalent (mg gallic acid/g extract) [21].

### 2.3. Cell Culture

VSMCs, purchased from Bio-Whittaker (San Diego, CA, USA), and RAW 264.7 cells, purchased from Korean Cell Bank (Seoul, Korea), were cultured Dulbecco's modified Eagel's medium (DMEM) supplemented with 10% heat-inactivated fetal bovine serum (FBS), 100 unit/mL penicillin, and 100 *μ*g streptomycin at 37°C in a humidified atmosphere containing 5% CO_2_. VSMCs were incubated in DMEM supplemented with 0.1% FBS for 24 h to synchronize them at G0 phase.

### 2.4. DPPH Scavenging Activity, Metal-Chelating Activity, and Lipid Peroxidation

DPPH scavenging activity and metal-chelating activity of TFW were measured as described previously [22, 23]. Effect of TFW on serum lipid peroxidation was determined by TBARS assay as described previously [22].

### 2.5. NO Production, iNOS, and COX-2 Expression in RAW 264.7 Cells

Effects of TFW on NO production, iNOS, and COX-2 expression in RAW 264.7 cells were determined as described previously [23]. Briefly, the cells were pretreated with TFW for 1 h followed by stimulation with 1 *μ*g/mL of lipopolysaccharide (LPS) for 24 h. Griess reagent was used to measure NO level in the supernatant. The absorbance of chromophore was measured at 540 nm. Similarly, LPS induced iNOS and COX-2 expression in RAW 264.7 cells were determined by Western blot.

### 2.6. Wound Healing Assay

The monolayer of confluent VSMCs was scratched with a yellow tip to create a migrating zone, followed by washing with phosphate buffered saline (PBS) to remove debris. TFW at different concentrations was pretreated for 1 h prior to stimulation of the cells with PDGF-BB (20 ng/mL) for 24 h. The distance of migrating zone was photographed in 0, 12, and 24 h by light microscope under a magnification of 100x [8].

### 2.7. Boyden's Chamber Assay

VSMC migration was performed with a modified Boyden's chamber assay. The lower face of polycarbonate membrane (Sigma Aldrich, USA) was coated with 2.5% gelatin in 1 M acetic acid. The cells were seeded at a density of 1 × 10^5^ cells/mL in 200 *μ*L of DMEM containing 0.1% bovine serum albumin (BSA) in the upper chamber, which was then immersed in lower chamber containing 600 *μ*L of DMEM with PDGF-BB (20 ng/mL) and TFW. The cells were allowed to migrate for 6 h. Afterwards, cells remaining on the inner membrane of the chamber were removed by cotton swabs. Migrated cells were fixed in 10% formalin and stained with hematoxylin and eosin. The membranes were washed and mounted on slides. The migrating cells were photographed by light microscope at the magnification of 200x [8].

### 2.8. Gelatin Zymography

VSMCs were pretreated with TFW for 1 h prior to stimulation with TNF-*α* (100 ng/mL) for 24 h. The enzymatic action of MMP-2 and MMP-9 in supernatant and cell lysate was determined by gelatin zymography as described previously [8]. Briefly, gels obtained after electrophoresis were washed in 2.5% Triton X-100 for 30 min and incubated at 37°C for 24 h in developing buffer. The gels were subsequently stained with coomassie brilliant blue and destained with destaining buffer. The zymograms were photographed. Proteolytic activity was detected as a white zone in the blue background.

### 2.9. Proliferation Assay

VSMC proliferation was assessed by using MTT assay as described previously [24]. VSMCs seeded in 96-well plates were pretreated with TFW for 1 h prior to stimulation of the cells with PDGF-BB (20 ng/mL) for additional 24 h. Then, MTT solution was added for 4 h followed by solubilization of formazan crystals in dimethyl sulfoxide (DMSO). The purple color thus formed was measured at 540 nm.

### 2.10. Western Blot Analysis

The protein expression of iNOS and COX-2 in RAW 264.7, activation of ERK, JNK, and FAK, and protein expression of MMP-9, RhoA, and Cdc42 in VSMCs were determined by Western blot analysis as described previously [24].

### 2.11. Statistical Analysis

SPSS (SPSS, Chicago, IL, USA) was used to perform statistical data analysis. All data were presented as mean ± standard deviation. Groups were compared by using one-way analysis of variance (ANOVA) followed by Duncan's* post hoc* test of multiple comparisons. *P* values ≤ 0.05 were considered statistically significant.

## 3. Results

### 3.1. Components of TFW

Dried fruits of* T. chebula*, obtained from Muan Province, Korea, were extracted with water as described under Materials and Methods to produce TFW dry powder. Total phenolic acid and total tannin content were found to be approximately 28.7% and 31.6%, respectively. The protein and polysaccharide contents of TFW were approximately 4.1%, and 9.1%, respectively. Because TFW contains abundant tannins, we assume that these tannins may be the main components responsible for the pharmacological effects of TFW.

### 3.2. TFW Scavenges Oxidation and Possesses Metal-Chelating Activity

**Figure 1:**
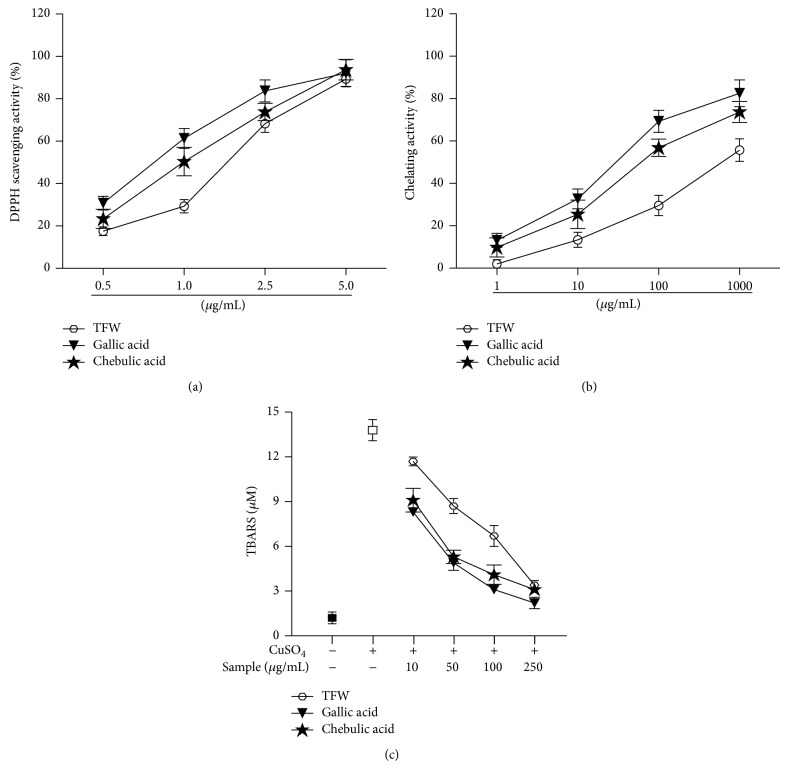
DPPH scavenging activity, NO scavenging activity, and metal chelation activity of TFW: (a) DPPH scavenging activity of TFW, gallic acid, and chebulic acid; values are expressed as mean ± standard deviation; *n* = 5. (b) Chelating activity of TFW, gallic acid, and chebulic acid; values are expressed as mean ± SD, *n* = 5. (c) Effect of TFW, gallic acid, and chebulic acid on serum lipid peroxidation; values are expressed as mean ± SD; *n* = 5.

Dose-response curve of DPPH scavenging activity of TFW, gallic acid, and chebulic acid is shown in Figure 1(a). Inhibitory concentration 50 (IC_50_) values of TFW, gallic acid, and chebulic acid were found to be 1.52, 0.85, and 1.1 *μ*g/mL, respectively.  Figure 1(b) shows the dose-response curve of metal-chelating activity of TFW and gallic acid. IC_50_ values of TFW, gallic acid, and chebulic acid for metal-chelating activity were 712.1, 33.4, and 75.4 *μ*g/mL, respectively. Nevertheless, when compared to EDTA (1 *μ*g/mL), the chelating activity of TFW or gallic acid or chebulic acid was found to be low. Furthermore, the concentration of malondialdehyde (MDA), a measure of serum lipid peroxidation, in the normal and the control groups was 1.2 ± 0.4 and 13 ± 0.7 *μ*M, respectively. TFW, gallic acid, and chebulic acid significantly reduced serum lipid peroxidation induced by CuSO_4_ in a dose dependent manner (Figure 1(c)).

### 3.3. TFW Inhibits iNOS, COX-2 Expression, and NO Production in RAW 264.7 Cells

**Figure 2:**
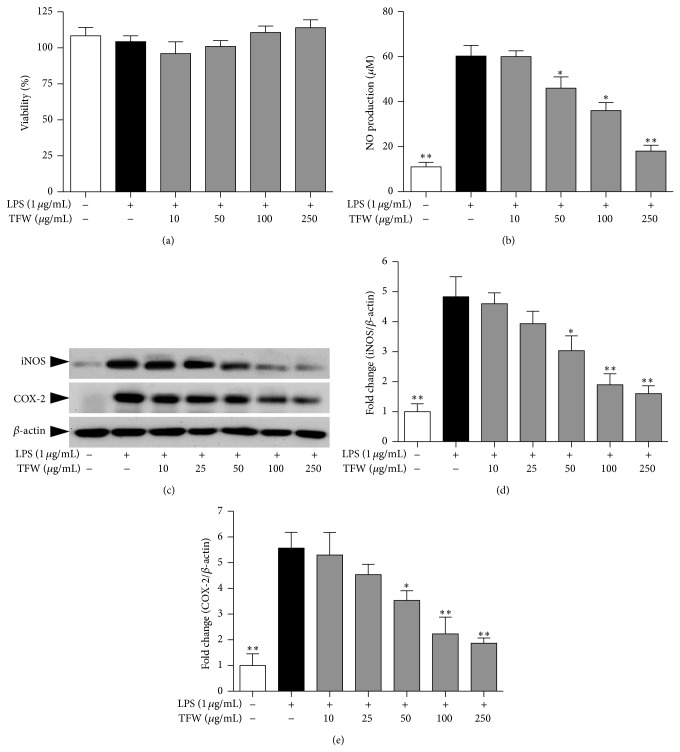
NO production, iNOS, and COX-2 expression in LPS stimulated RAW 264.7. RAW 264.7 cells were pretreated with TFW for 1 h followed by stimulation with LPS for additional 24 h. (a) The cell viability was determined using MTT assay. (b) NO production was measured using Griess reagent. (c) The expression of iNOS and COX-2 was determined by Western blot. *β*-actin was used for normalization. (d) Normalized quantitative data for iNOS expression. (e) Normalized quantitative data for COX-2 expression. Values are expressed as mean ± SD; *n* = 4. ^*^ *P* < 0.05 and ^**^ *P* < 0.01 versus control (LPS alone).

The anti-inflammatory effect of TFW was determined in lipopolysaccharide (LPS) induced RAW 264.7 cells. TFW up to 250 *μ*g/mL did not show any toxicity in normal cells (Figure 2(a)). Stimulation of the cells with LPS for 24 h increased the production of NO (56.6 ± 2.6 *μ*M). TWF dose dependently decreased LPS induced NO production (Figure 2(b)). Similarly, the expression of inflammatory enzymes COX-2 and iNOS were induced by LPS. TWF significantly decreased the expression of both iNOS and COX-2 (Figures 2(c)–2(e)).

### 3.4. TFW Inhibits VSMC Migration

**Figure 3:**
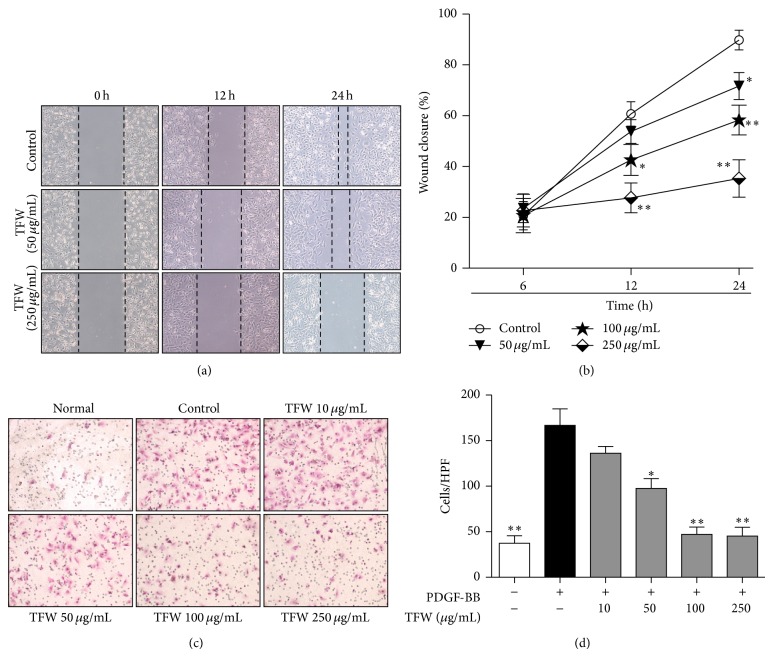
Effect of TFW on VSMC migration. (a) Subconfluent VSMCs were scraped off with a pipette tip to induce wounds and then the cells were treated with TFW for 1 h followed by PDGF-BB treatment. Images of wounded area were captured immediately (time 0) and 6, 12, and 24 h after injury. (b) Finally wound closure was quantified as percentage of the initial wound area that had been recovered with VSMCs. Values are expressed as mean ± SD (*n* = 5). ^*^ *P* < 0.05 and ^**^ *P* < 0.01 versus control (PDGF-BB alone); original magnification 200x. (c) The cells were allowed to migrate in the presence of PDGF-BB and the indicated concentrations of TFW for 6 h. After staining with eosin and hematoxylin, the migrated cells were counted (d) in 5 high power fields (200x). Values are expressed as mean ± SD (*n* = 5). ^*^ *P* < 0.05 and ^**^ *P* < 0.01 versus control (PDGF-BB alone).

The effects of TFW on VSMC migration as evaluated by using a wound healing assay and Boyden chamber are as shown in Figure 3. TFW dose dependently suppressed PDGF-BB induced VSMC wound healing for 6, 12, and 24 h after injury (Figure 3(b)). TFW at 100 and 250 *μ*g/mL showed the significant inhibition of healing (approximately 30% and 56% compared with control, resp.) at 12 h. The magnitude of inhibition remained suppressed with 50, 100, and 250 *μ*g/mL of TFW (approximately 20%, 35%, and 60% compared with control, resp.) at 24 h. Next, we evaluated VSMC migration using a modified transwell apparatus. The cellular migration was induced by PDGF-BB that increased the basal migration of VSMC by 4.4-fold compared to PDGF-BB nontreated cells. TFW at 50, 100, and 250 *μ*g/mL inhibited PDGF-BB-stimulated VSMC migration by 45%, 74%, and 75%, respectively (Figure 3(d)).

### 3.5. TFW Inhibits MMPs Expression and FAK Activation to Attenuate VSMC Migration

**Figure 4:**
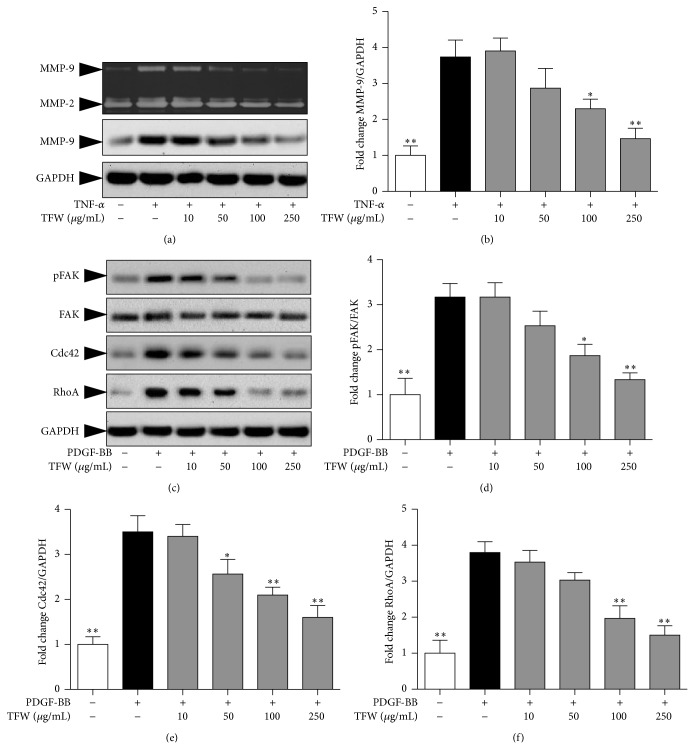
Effect of TFW on MMP-2 and MMP-9 expression. VSMCs were pretreated with TFW for 1 h followed by stimulation with TNF-*α* for 24 h. (a) The proteolytic activity of MMP-2 and MMP-9 in conditioned medium and cell lysate was measured by gelatin zymography. The protein expression of MMP-9 was determined by Western blot. GAPDH was used for normalization. (b) Normalized quantitative data for MMP-9 protein expression. (c) Serum starved VSMCs were pretreated with indicated concentrations of TFW for 1 h followed by stimulation with PDGF-BB for additional 15 min. The cell lysates were assayed for protein expression of pFAK, FAK, RhoA, and Cdc42. (d) Normalized quantitative data for FAK activation. (e) Normalized quantitative data for Cdc42 expression. (f) Normalized quantitative data for RhoA expression. Values are expressed as mean ± SD; *n* = 3. ^*^ *P* < 0.05 and ^**^ *P* < 0.01 versus control (PDGF-BB alone).

VSMC migration involves both degradation of ECM by MMPs and cytoskeletal remodeling, which is regulated by various growth factors and cytokines [8]. MMP-2 and MMP-9 have been reported to degrade ECM thereby facilitating VSMC migration. We used TNF-*α* to induce MMP-9 expression. As shown in Figure 4(a), stimulation of VSMCs with TNF-*α* for 24 h enhanced the proteolytic activity and protein expression of MMP-9 while MMP-2 was constitutively secreted. The pretreatment of TFW strongly inhibited MMP-9 proteolytic activity and protein expression (Figures 4(a)-4(b)). Upon cells adhesion to ECM, FAK becomes activated through tyrosine phosphorylation, notably at Tyr397 [25]. We next examined if TFW affected PDGF-BB-induced FAK phosphorylation. As shown in Figures 4(c)-4(d), TFW inhibited PDGF-BB-induced FAK phosphorylation, whereas the total FAK remained unchanged. FAK phosphorylation follows RhoA and Cdc42 activation, which promotes cytoskeletal remodeling leading to the formation of stress fibers and filopodia [9]. As shown in Figures 4(c), 4(e)-4(f), TFW significantly inhibited RhoA and Cdc42 expression.

### 3.6. TFW Inhibits VSMC Proliferation

**Figure 5:**
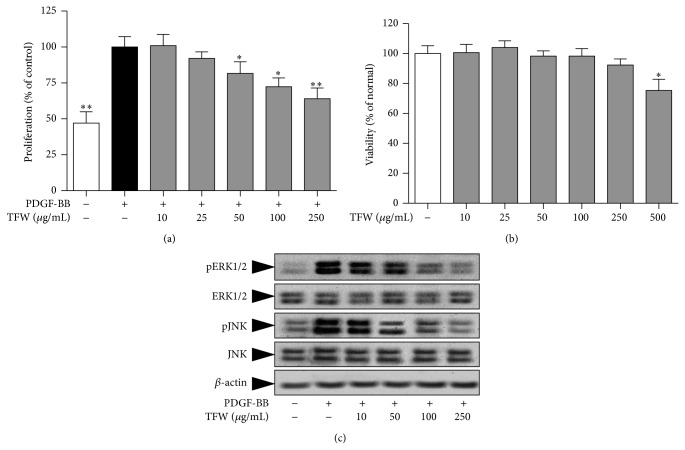
Effect of TFW on PDGF-BB induced VSMC proliferation. (a) Serum starved VSMCs were pretreated with indicated concentrations of TFW followed by stimulation with PDGF-BB (20 ng/mL) for 24 h. Cell proliferation was measured by MTT assay and values are expressed as mean ± SD; *n* = 6. ^*^ *P* < 0.05 and ^**^ *P* < 0.01 versus control (PDGF-BB alone). (b) Serum starved VSMCs were treated with indicated concentrations of TFW without stimulation for 24 h. Cell viability was measured by MTT assay. (c) Serum starved VSMCs were pretreated with indicated concentrations of TFW for 1 h followed by stimulation with PDGF-BB for additional 15 min. The cell lysates were assayed for protein expression of p-ERK1/2, ERK1/2, pJNK, and JNK. Values are expressed as mean ± SD; *n* = 6. ^*^ *P* < 0.05 versus normal (untreated).

PDGF-BB, a chemotactic and mitogenic factor, gets upregulated in atherosclerosis and restenosis [2]. As shown in Figure 5(a), stimulation of VSMCs with PDGF-BB increased the cell number after 24 h. The treatment of TFW dose dependently inhibited VSMC proliferation induced by PDGF-BB. TFW at concentrations of 50, 100, and 250 *μ*g/mL significantly reduced the proliferation rate to 81.6%, 72.3%, and 64% of the control (PDGF-BB treated without TFW). To confirm that the inhibitory effects were not due to toxicity or damage to the cells, various concentrations of TFW were treated in nonstimulated cells for 24 h. TFW had no effect on the basal level of cell viability. However, TFW at a concentration of 500 *μ*g/mL possessed cytotoxicity (Figure 5(b)). So, this concentration was not included in further studies.

### 3.7. TFW Inhibits MAPK Signaling to Suppress VSMC Proliferation

MAPKs, which have different functions and can crosstalk at several levels to initiate the transcription of several immediate early genes required for proliferation, are activated in response to inflammatory and atherogenic stimuli including PDGF-BB [4]. Serum deprived VSMCs were stimulated with PDGF-BB for 15 min and the activation of ERK1/2 and JNK was determined by Western blot. As shown in Figure 5(c), stimulation with PDGF-BB overexpressed pERK1/2 and p-JNK. The pretreatment of TFW inhibited ERK1/2 and JNK phosphorylation.

## 4. Discussion

In this study, we showed that TFW possesses strong antioxidative activity. Also, TFW inhibited proinflammatory mediators, iNOS and COX-2 expression in RAW267.4 cells. Furthermore, TFW attenuated PDGF-BB induced VSMC proliferation and migration. Oxidative stress plays an important role in the progression of atherosclerosis. The reactive oxygen and nitrogen species produced by vascular cells contributes to endothelial dysfunction and plaque disruption [26]. ROS can activate a complex cascade of signal transduction pathways that are highly associated with inflammation, immunity, and atherosclerosis [27]. TFW possessed strong DPPH scavenging activity with the possibility to act as primary antioxidants. Lipid peroxidation is catalyzed by metal ions to alkoxy and peroxy radicals and TFW strongly inhibited metal-induced serum lipid peroxidation. Moreover, TFW possessed strong iron-binding capacity, suggesting that its inhibitory role in serum lipid peroxidation might be due to its chelating effect. Sun et al. reported that chebulic acid efficiently scavenged free radical and significantly reduced the tert-butyl hydroperoxide- (t-BHP-) induced cell cytotoxicity and intracellular ROS in rat hepatocytes [28]. The strong metal-chelating activity of TFW might be due to higher tannins content in it as they possess more structural features for complexing metal ions [29]. Several recent studies demonstrated the therapeutic benefits of chelators in metal-catalyzed oxidative stress-mediated chronic diseases including diabetes and cardiovascular diseases [30].

Inflammation has been considered as the primary process of atherosclerosis [11, 12]. We determined iNOS and COX-2 expression and NO production in LPS stimulated RAW 264.7 cells as biomarkers of inflammatory response. The circulating monocytes get recruited and differentiated into macrophages in response to monocyte chemoattractant protein (MCP-1) [10]. Lee et al. reported that chebulic acid rich* T. chebula* inhibited monocyte adhesion to endothelial cells by inhibiting ICAM-1 expression suggesting a potential role of TFW in inhibiting circulating monocytes recruitment into the intima [31]. It is interesting that a significant correlation has been found between the degree of macrophage infiltration and endothelial ICAM-1 expression in atherosclerotic lesions [32]. Activation of macrophages in the intima results in iNOS and COX-2 synthesis. NO production due to catalytic activity of iNOS results in subsequent formation of peroxynitrite that can lead to LDL oxidation [12, 13]. Also, excessive COX-2 expression plays a key role in inflammatory disorders including atherosclerosis and inhibition of COX-2 expression is a therapeutic target for inflammatory diseases [11]. TFW inhibited iNOS and COX-2 expression and NO production, suggesting their beneficial role in the management of inflammation.

Since VSMC proliferation and migration are one of the leading factors in the progression of atherosclerosis and restenosis [3], inhibition of VSMC proliferation and migration represents an important therapeutic strategy for the prevention of atherosclerosis and restenosis. Various growth factors, including PDGF-BB and TNF-*α*, are upregulated in atherosclerosis [1, 2]. In our* in vitro* model of VSMC proliferation, the treatment of TFW dose dependently inhibited PDGF-BB induced VSMC proliferation. The molecular mechanisms that regulate PDGF-BB-mediated responses have been intensively studied in recent years. PDGF-BB activate MAPKs known as JNK, P38, and ERK, which can crosstalk at several levels to initiate the transcription of several immediate early genes involved in cell cycle that drive cellular proliferation and growth in mammalian cells [4]. Our study showed that treatment of TFW strongly inhibited PDGF-BB-induced ERK1/2 and JNK phosphorylation. Chebulagic acid from* T. chebula* resulted in cell cycle arrest by increasing the expression of cell cycle dependent kinase (CDK) inhibitor p27 in retinoblastoma cells [33]. Moreover, ERK/2 inhibitor caused cell cycle arrest to prevent VSMC proliferation by inhibiting cyclins and CDKs expression [24]. Altogether, the inhibitory effect of TFW on VSMC proliferation could be due to cell cycle arrest via inhibition of ERK1/2 activation. VSMC migration would require, at a minimum, (a) a chemoattractant to direct their movement toward the intima, (b) the ability to breach and transverse the ECM barriers, and (c) the activation of the cellular machinery for cell movement in response to the chemoattractant [34]. We used PDGF-BB as a chemoattractant, since considerable evidence supports a role of PDGF-BB in VSMC migration* in vitro* and* in vivo* [5]. TFW strongly inhibited PDGF-BB induced VSMC migration. The second requirement for VSMC migration is to degrade ECM, which is mediated by MMPs [6]. The basal level of MMP-9 in VSMCs is low and its expression can be induced by TNF-*α* [20, 22]. TFW markedly decreased TNF-*α* induced MMP-9 enzymatic action and protein expression while MMP-2 was constitutively secreted. To explore the possible underlying MMP-9 inhibitory mechanism of TFW, we attempted to examine the effect of TFW in JNK activation. It has been well reported that the phosphorylation of transcription factor c-Jun by JNK results in translocation of the Fos-Jun complex to the nucleus and activation of MMP-9 promoter [35]. We previously showed that ERK inhibitor weakly and JNK inhibitor strongly inhibited the enzymatic action and protein expression of MMP-9 [8]. We found that TFW inhibited JNK activation, which might in turn inhibit MMP-9 expression to inhibit VSMC migration. Another possible reason for inhibition of enzymatic action of MMP-9 is chelation of Zn^+2^ in the catalytic site of MMPs by TFW, thereby causing inactive protease. The third requirement for VSMC migration is cytoskeleton remodeling mediated by ECM or PDGF-BB, which drives the cellular machinery through FAK activation. Activated FAK has the ability to transduce signals downstream by interacting with multiple signaling molecules, including small GTPases [36]. The Rho GTPases including RhoA and Cdc42 are involved in mediating cytoplasmic actin filament organization that occurs in migrating cells stimulated by chemoattractants [37, 38]. TFW inhibited PDGF-BB induced FAK phosphorylation and subsequent small GTPases, RhoA and Cdc42, expression to inhibit cytoskeleton remodeling. These findings strongly suggest that TFW is a potent inhibitor of VSMC migration.

## 5. Conclusions

In conclusion, we demonstrated that TFW scavenged oxidation and inhibited the production of proinflammatory mediators in LPS stimulated RAW 264.7 cells. Similarly, TFW attenuated PDGF-BB induced VSMC proliferation and migration. The anti-proliferative effect of TFW appeared to be dependent on inhibition of ERK1/2 activation. Similarly, the antimigratory effect of TFW is found to be dependent on its inhibitory action on MMP-9 expression and FAK activation. Based on our current findings, we speculate that TFW could be used as a therapeutic agent in the prevention of atherosclerosis. However, further studies are needed to identify the possible active compounds that are possibly responsible for the antiatherosclerotic properties of TFW, which may provide an opportunity to develop a new class of antiatherosclerotic drugs.
